# "Enemies of the People?" Public Health in the Era of Populist Politics: Comment on "The Rise of Post-truth Populism in Pluralist Liberal Democracies: Challenges for Health Policy"

**DOI:** 10.15171/ijhpm.2017.46

**Published:** 2017-04-15

**Authors:** Martin McKee, David Stuckler

**Affiliations:** ^1^ECOHOST, Department of Health Services Research and Policy, London School of Hygiene and Tropical Medicine, London, UK.; ^2^Dipartimento di Analisi delle Politiche e Management Pubblico, Università Bocconi, Milan, Italy.

**Keywords:** Public Health, Advocacy, Political Economy, Fake News

## Abstract

In this commentary, we review the growth of populist politics, associated with exploitation of what has been termed fake news. We explore how certain words have been used in similar contexts historically, in particular the term "enemy of the people," especially with regard to public health. We then set out 6 principles for public health professionals faced with these situations. First, using their epidemiological skills, they can provide insights into the reasons underlying the growth of populist politics. Second using their expertise in modelling and health impact assessment, they can anticipate and warn about the consequences of populist policies. Third, they can support the institutions that are necessary for effective public health. Fourth they can reclaim the narrative, rejecting hatred and division, to promote social solidarity. Fifth, they can support fact checking and the use of evidence. Finally, they should always remember the lessons of history, and in particular, the way that public health has, on occasions, collaborated with totalitarian and genocidal regimes.

In the accompanying paper, Speed and Mannion discuss the rise of populist politics in liberal democracies, and in particular, the role played by fake news.^1^ In several Western countries, there is growing alarm about the growing appeal of politicians who have, in effect, torn up the rulebook. Showing a complete contempt for the institutions created over decades to promote good governance, and for enlightenment concepts of evidence, they have used “the will of the people,” at least as interpreted by themselves, to pursue their personal objectives.^2^ Most attention has, inevitably, focused on Donald Trump, who uses Twitter to scatter unfounded allegations in all directions. Conventionally, American presidents have been informed by carefully analysed briefings, including a discussion of the strengths and weaknesses of the evidence on which they are based. In contrast, this president appears to obtain much of his information from a few selected television channels. Once he has tweeted what he has heard to his 26 million followers, no matter how ridiculous the claims, they are picked up in the echo chamber of social media and, in those same channels that he watches, thereby reinforcing his self-belief.^3^

Efforts by large parts of the mainstream media to put the record straight, for example by pointing out, in news headlines, that the president’s claims are totally unsubstantiated, have little impact. What is important is that he has created a narrative that people believe. Once that has been achieved, it is very difficult to change. Previous research shows how even authoritative corrections often have the effect of reinforcing the initial false belief among those who have strongly held views.^4^ Moreover, to encourage this process, President Trump has engaged in sustained attacks against some of the leading, and most authoritative media outlets, including, as he describes it, the “failing” New York Times. Those newspapers, along with the courts that have refused to uphold his unconstitutional executive orders, are labelled as part of the liberal elite conspiring against the will of the people. Worse, they are, in President Trump’s view, the “enemies of the people.”

Yet the problems are not confined to the United States. Populist policies, often based on outright lies, have been gaining support in other countries, although in some, such as the Netherlands and Germany, there is some evidence that people are now recoiling in horror from what they see in the United States. More worrying is the United Kingdom, where a tabloid newspaper also used the term “enemies of the people” to describe the three High Court judges who ruled that the British government was required to submit its decision to leave the European Union for Parliamentary approval. Remarkably, in a country that takes pride in its democratic institutions, based on “the mother of parliaments,” the government was seeking to exclude Parliament from having a voice on the greatest constitutional change in four decades.

So, given the growing use of the term “enemies of the people,” it seems timely to reflect on its origins. Although the earliest uses seem to date back to Roman times, it gained widespread currency during the French Revolution, when it was used to describe those who criticise the reign of terror. Robespierre claimed that the revolution “ne doit aux ennemies du people que la mort [owed nothing to the enemies of the people but death]” and many of those he was describing would ultimately fall victim to the terror.^5^ Ironically, given later developments, one of the offences punishable by death in a 1794 law on the “enemies of the people” was “spreading false news to divide or trouble the people.”

Interestingly, the next incarnation of this term placed health centre stage, quite literally. In his 1882 play, “An enemy of the people,” the Norwegian playwright Henrik Ibsen describes how Dr. Stockmann, the municipal doctor in a small town, has discovered that the public baths are contaminated and people are falling ill.^6^ This has major implications as they bring much-needed revenue to the town. He must choose whether to remain silent or to speak out and alienate the vast majority of the townspeople. He speaks out and, in the final scene, he is evicted from his home, whose windows have just been smashed by the crowds. Yet he sticks to his principles, declaring that “the strongest man is he who stands alone.”

In marked contrast to Ibsen’s use of the term as a mark of courage, Lenin reverted to the French revolutionary usage. He justified a 1917 decree dissolving a constitutional political party on account of it being full of “enemies of the people,” who were to be arrested and brought in front of a revolutionary court. Under Stalin being identified as this group, now extended to include “enemies of the workers” or “enemies of the proletariat,” was enough to allow people to be imprisoned, sent into exile, or executed.^7^ In Germany, the term was taken up by Joseph Goebbels, when he described Jews as “a sworn enemy of the German people.”^7^ Consequently, those who use these words now should understand their historical significance.

Seen in this context, perhaps those of us who seek to protect and promote health using evidence-based policies should see the label of “enemy of the people” as one that we should aspire to, in the noble tradition of Dr. Stockmann. Certainly, there have been many advocates for public health that have spoken truth to power, challenging powerful vested interests. Many would not have seen themselves as part of the public health community, such as Rachel Carson who, in her book Silent Spring,^8^ drew international attention to the threat from pesticide residues. Others would have, including those who have courageously exposed the unethical, and in some cases, illegal activities of the tobacco industry. Yet others, and especially those with their roots in medicine, have included committed public health professionals. These include the International Physicians for the Prevention of Nuclear War, winners of the Nobel Peace Prize in 1985.

All these groups recognise their duty to speak truth to power. Those with epidemiological skills can make the invisible visible, drawing connections that reveal otherwise unseen patterns of disease, describing hidden inequalities within society, and giving voice to those who are marginalised or oppressed. But they must realise that, in the climate prevailing in many countries today, this will place them firmly within the category of enemies of the people.

So what should public health do at a time when the populist politicians in the ascendant in many countries reject scientific evidence, replacing it with fake news? How to they respond to those who sow divisions in society, employing the long-established tactic of divide and rule? These are not hypothetical questions. The new American Secretary for the Environment has questioned whether global warming really is man-made. President Trump has floated the now totally discredited suggestion that vaccines cause autism.

First, the public health community can provide insights into how populist politicians have become so powerful. Some, as Speed and Mannion note,^1^ is due to the growth of identity politics, as established populations react against what they see as the incursion of those from other cultures.^9^ Yet there is also growing evidence that this process is encouraged by growing inequalities in society, with some groups feeling left behind in a world that they increasingly struggle to recognise. As an analysis for the Economist noted, one of the strongest predictors of a shift in voting behaviour towards Trump was a composite measure of poor health.

Second, drawing on methods such as modelling, natural experiments, and health impact assessment, it can highlight the consequences of policies promoted by these politicians. Many policies promoted by populist politicians will damage their strongest supporters. For example, politicians supporting Brexit look forward to the opportunity to tear up “red tape,” passing over the inconvenient detail that this will remove the employment and safety rights of their strongest supporters. The imaginative use of graphics can be very powerful, as in a recent Los Angeles Times picture showing how many who would lose most from repeal of the Affordable Care Act were in countries that had voted for Trump.^10^

Third, public health can support the institutions on which government, and especially health policy depends. Some populist politicians seek untrammelled power, unchecked by the courts, exemplified by the British government’s failed legal attempt to block Parliament from debating Brexit. Yet independent courts have consistently safeguarded public health in the face of powerful vested interests.^11^ It is also important to support those institutions involved in collecting and analysing the data on which epidemiology depends, especially when politicians use the excuse of financial pressures to reduce data collection, as the previous Canadian government did with the long form element of the census.^12^ The attack on the highly respected US Congressional Budget Office by officials from the Trump administration, following its publication of a report showing that an estimated 24 million Americans would lose coverage under the Republican’s health reform plan, exemplifies the threat.^13^

Fourth, public health should reclaim the narrative. Populist politicians succeed where they achieve ownership over language. Every problem is blamed on the “other,” typically ethnic or religious minorities. In the United Kingdom, supporters of Brexit recast the very visible problems facing the National Health Service as being due to high levels of use by migrants when, in fact, the service depended on large numbers of migrant workers to function.^14^ Particular care is needed with words such as “terrorist” which, in the United States and some other countries, is being redefined to include only perpetrators who are Muslims.^15^ The results can be seen when news networks delay labelling a mass shooting as “terrorist” until they have identified the attacker.^16^ However, when such attacks occur, there is a need for particular care as those advocating repressive measures will seek to exploit them. Sometimes, public health professionals will be drawn into responses, through their roles in emergency planning. They must demand that any measures taken are evidence-based and proportionate.^17^

Fifth, public health professionals can support fact checking organisations, offering their expertise to analyse claims by politicians and other commentators. The public health community has extensive experience in revealing how vested interests, especially the tobacco industry^18^ but now joined by the alcohol^19^ and soft drinks industries,^20^ use seemingly independent front groups to disseminate their messages. The same approach can be used to expose the forces behind those groups promoting hatred and division.

Finally, the public health community must never forget the lessons of history. Physicians played a prominent role in many of the most appalling actions of the Third Reich, not just in the notorious experiments of those such as Joseph Mengele, but in many other ways, including providing the intellectual underpinning of eugenic and racist policies.^21-23^ It has been estimated that up to two-thirds of physicians were affiliated, in some way, with the Nazi party and related institutions,^24^ while the medical profession was complicit in the prohibition of Jewish doctors working for the state or the sickness funds. Arthur Guett, the Reich Interior Ministry’s public health director described the “supreme duty of the nation state to grant life and livelihood only to the healthy and hereditarily sound portion of the people.”^21^ In occupied Poland the Nazi governor sought to prevent transmission of tuberculosis by executing infected Poles.^25^ And it was not only Nazi Germany. Public health professionals facilitated programs to sterilise those with learning difficulties in many other countries, including the United States and Scandinavia.^26^ History is also a reminder of the danger of complacency, with clear signs of political danger being dismissed as alarmism, as when the New York Times, concluded that, after his release from prison in 1924, Hitler “was no longer to be feared” and it was expected that he would “retire to private life.”^27^

We are living in dangerous times as post-truth populism is propelling into power politicians who are both dangerous and grossly incompetent. Yet there is hope. We have been here before but this time we must ensure that public health is on the right side.

*Note: This commentary draws on a plenary lecture given by MM at the World Congress of Public Health in Melbourne, April 2017*.

## Ethical issues

Not applicable.

## Competing interests

The authors declare that they have no competing interests.

## Authors’ contributions

Both authors contributed equally to the writing of this paper.

## Authors’ affiliations

^1^ECOHOST, Department of Health Services Research and Policy, London School of Hygiene and Tropical Medicine, London, UK. ^2^Dipartimento di Analisi delle Politiche e Management Pubblico, Università Bocconi, Milan, Italy.
